# The interplay between metabolic alterations, diastolic strain rate and exercise capacity in mild heart failure with preserved ejection fraction: a cardiovascular magnetic resonance study

**DOI:** 10.1186/s12968-018-0511-6

**Published:** 2018-12-24

**Authors:** Masliza Mahmod, Nikhil Pal, Jennifer Rayner, Cameron Holloway, Betty Raman, Sairia Dass, Eylem Levelt, Rina Ariga, Vanessa Ferreira, Rajarshi Banerjee, Jurgen E. Schneider, Christopher Rodgers, Jane M. Francis, Theodoros D. Karamitsos, Michael Frenneaux, Houman Ashrafian, Stefan Neubauer, Oliver Rider

**Affiliations:** 1 Division of Cardiovascular Medicine, Radcliffe Department of Medicine, University of Oxford Centre for Clinical Magnetic Resonance Research (OCMR), University of Oxford, John Radcliffe Hospital, Headley Way, Oxford, OX3 9DU UK; 2 National University of Malaysia Medical Centre, Kuala Lumpur, Malaysia; 3 Divisions of Experimental Therapeutics and Cardiovascular Medicine, Radcliffe Department of Medicine, Oxford, UK; 4 1st Department of Cardiology, Aristotle University, Thessaloniki, Greece; 5 Department of Medicine, John Radcliffe Hospital, Oxford, UK; 6 Wolfson Brain Imaging Centre, University of Cambridge, Cambridge, UK; 7 Norwich Medical School, Bob Champion Research and Education Building, James Watson Road, University of East Anglia Norwich Research Park, Norwich, NR4 7UQ UK

**Keywords:** Cardiovascular magnetic resonance, Spectroscopy, Diastolic strain rate, Heart failure, Steatosis, Maximal oxygen consumption

## Abstract

**Background:**

Heart failure (HF) is characterized by altered myocardial substrate metabolism which can lead to myocardial triglyceride accumulation (steatosis) and lipotoxicity. However its role in mild HF with preserved ejection fraction (HFpEF) is uncertain. We measured myocardial triglyceride content (MTG) in HFpEF and assessed its relationships with diastolic function and exercise capacity.

**Methods:**

Twenty seven HFpEF (clinical features of HF, left ventricular EF >50%, evidence of mild diastolic dysfunction and evidence of exercise limitation as assessed by cardiopulmonary exercise test) and 14 controls underwent ^1^H-cardiovascular magnetic resonance spectroscopy (^1^H-CMRS) to measure MTG (lipid/water, %), ^31^P-CMRS to measure myocardial energetics (phosphocreatine-to-adenosine triphosphate - PCr/ATP) and feature-tracking cardiovascular magnetic resonance (CMR) imaging for diastolic strain rate.

**Results:**

When compared to controls, HFpEF had 2.3 fold higher in MTG (1.45 ± 0.25% vs. 0.64 ± 0.16%, *p* = 0.009) and reduced PCr/ATP (1.60 ± 0.09 vs. 2.00 ± 0.10, *p* = 0.005). HFpEF had significantly reduced diastolic strain rate and maximal oxygen consumption (VO_2_ max), which both correlated significantly with elevated MTG and reduced PCr/ATP. On multivariate analyses, MTG was independently associated with diastolic strain rate while diastolic strain rate was independently associated with VO_2_ max.

**Conclusions:**

Myocardial steatosis is pronounced in mild HFpEF, and is independently associated with impaired diastolic strain rate which is itself related to exercise capacity. Steatosis may adversely affect exercise capacity by indirect effect occurring via impairment in diastolic function. As such, myocardial triglyceride may become a potential therapeutic target to treat the increasing number of patients with HFpEF.

## Background

Almost half of all patients who present with clinical features of heart failure (HF) have preserved left ventricular (LV) ejection fraction (HFpEF). Its prevalence is on the rise, representing a major burden for health care services [1]. These patients are often elderly, female with multiple co-morbidities such as hypertension and obesity, and typically show a non-dilated LV, concentric remodelling and abnormal diastolic function [2]. The diagnostic criteria of HFpEF is based on clinical features of HF and normal LV ejection fraction (EF) together with evidence of diastolic dysfunction, LV hypertrophy, left atrial (LA) enlargement and raised plasma brain natriuretic peptides (BNP) according to the current European Society of Cardiology (ESC) guidelines [3].

However, the current criteria focus on patients with more advanced stages of the diseases leaving a large proportion of patients with mild diastolic dysfunction and limiting dyspnoea which may be overlooked in daily clinical practice. In addition, the vast majority (90%) of elderly patients with isolated moderate/severe diastolic dysfunction and normal LVEF not diagnosed to have HF have limiting dyspnoea [4]. Furthermore, nearly a third of asymptomatic patients with diastolic dysfunction develop symptoms of breathlessness, oedema or fatigue over 2 years [5] and this progression is accompanied by a substantial decrease in survival rate [6]. As a result, there is a real need for novel and, effective therapeutic targets to improve the management of the increasing number of patients with HFpEF.

Myocardial metabolism is a promising therapeutic target in HF [7]. HF is characterized by impaired myocardial energetics and altered myocardial substrate metabolism with a switch in fatty acid (FA) oxidation towards glucose oxidation for adenosine triphosphate (ATP) generation. This metabolic substrate shift may be a consequence of glucose being energetically more efficient (lower oxygen usage) than fatty acids [7, 8]. Accompanying this switch is an imbalance between FA uptake (which continues to be high) and FA oxidative metabolism (which is reduced), leading to intracellular cardiac lipid accumulation. This accumulation provides a source for non-oxidative metabolism to diacylglycerol and ceramide, potentially resulting in lipotoxicity, apoptosis and cardiac dysfunction [8–10].

Proton (^1^H) cardiovascular magnetic resonance (CMR) spectroscopy (CMRS) allows the non-invasive measurement of myocardial lipid (triglyceride) content [11], and by using this technique, we and others have demonstrated that cardiac steatosis occurs in several situations that are characterised by diastolic dysfunction; aortic stenosis, metabolic syndrome, obesity and type 2 diabetes mellitus [12–14]. Whilst steatosis [15] and reduced myocardial energetics [16] have both been shown to be present in HFpEF, metabolic characterization assessing both myocardial triglyceride (MTG) and energetics within the same cohort of patients with HFpEF and their relationship with cardiac function and exercise capacity have not previously been explored. The focus of this study was to investigate MTG content and energetics in patients with mild HFpEF and their relationship with cardiac function and exercise capacity.

CMR feature-tracking (CMR-FT) is a technique recently developed that can quantify strain and diastolic strain rate based on standard CMR cine balanced steady state free precession (bSSFP) images independent of additional sequences, with considerably reduced post processing time, and has been shown to have good agreement with CMR tissue tagging [17]. Although CMR tissue tagging is the gold standard for measuring cardiac strain, increased scanning time due to acquisition of additional tagging sequences and processing time limits its routine use [17]. We used multi-parametric cine CMR and multinuclear CMRS (^1^H and ^31^P) to investigate metabolic changes in HFpEF, and their relationships with diastolic function (diastolic strain rate) and exercise capacity, as measured by maximal oxygen consumption (VO_2_ max).

## Methods

### Study population

A group of 27 patients with HFpEF defined by the presence of symptoms or signs of HF, a non-dilated LV with LVEF ≥50% and evidence of abnormal diastolic function on Doppler echocardiography were prospectively enrolled between 2011 and 2018 [18, 19]. Additionally, we performed cardiopulmonary exercise testing (CPET) to confirm their exercise limitation by measuring VO_2_ max (VO_2_ at peak exercise). Patients were enrolled if their VO_2_ max was < 80% predicted for age and gender, with a pattern of gas exchange that would indicate a cardiac cause of exercise limitation. Patients who had diabetes mellitus, uncontrolled hypertension, significant valvular disease, previous myocardial infarction, coronary revascularization, previous cardiac surgery, contraindications to CMR or estimated glomerular filtration rate < 30 ml/min were excluded. Fourteen age, gender and body mass index (BMI) matched healthy controls without a history of heart disease, diabetes, hypertension or dyslipidaemia were also recruited. They were identified from the local population by word of mouth and poster advertisements around hospital and university. Controls were included if they were 65 years or older, asymptomatic, not on any medications and had no cardiac abnormalities detected on electrocardiogram (ECG) or CMR. All study participants fasted for at least 6 h prior to CMR and CMRS and were scanned at around the same time of the day.

### Study protocol

All study participants underwent resting transthoracic echocardiography, ^31^P-CMRS (*n* = 20 for HFpEF and *n* = 10 for controls) and ^1^H-CMRS in addition to the standard CMR imaging. All HFpEF patients and a subgroup of healthy controls (*n* = 9) underwent CPET.

### ^1^H cardiovascular magnetic resonance spectroscopy

Myocardial ^1^H spectra were obtained from the mid interventricular septum as previously described [11]. In short, spectroscopic acquisitions were performed using ECG trigger at end-expiration to minimize motion artefacts. Water-suppressed spectra were acquired to measure cardiac lipid, and spectra without water suppression were acquired and used as an internal standard. Spectroscopic stimulated echo (STEAM) sequence with an echo time (TE) of 10 ms was used. Five to six water-suppressed scans (5 averages each; repetition time (TR) of at least 2 s) were acquired in mid-diastole in a series of single breath-holds of about 10 s each. Next, a water spectrum (3 averages; TR of at least 4 s) was acquired in a single breath-hold to use as internal reference. Spectra were analysed using a custom Matlab (Mathworks, Natick, Massachusetts, USA) implementation of AMARES (Advanced Method of Accurate, Robust and Efficient Spectroscopic) and the AMARES algorithm in jMRUI (Java-based Magnetic Resonance User Interface) as previously described [11]. Myocardial triglyceride content was calculated as a percentage relative to water: (signal amplitude of lipid/signal amplitude of water) × 100%.

### ^31^P cardiovascular magnetic resonance spectroscopy

^31^P-CMRS was performed to obtain PCr/ATP ratio from a voxel placed in the mid-ventricular septum, with the subjects lying prone with their heart over the centre of the ^31^P heart/liver coil in the magnet isocentre as previously described [20]. ^31^P-CMRS post processing analysis was performed using a custom Matlab analysis tool, as previously described [20]. Coil positioning was confirmed or adjusted with the use of proton localization images as previously described. Spectra were acquired using acquisition-weighted 3D chemical shift imaging (CSI) [21], with 10 averages in the centre of K-space. The TR was 720 ms and the acquisition duration was 8 min [22].

### Cardiovascular magnetic resonance imaging

Cine CMR images were acquired for cardiac volume analysis using a 3 T CMR system (Trio, Siemens Healthineers, Erlangen, Germany) using bSSFP cine imaging as previously described [23]. Analysis of cardiac volumes, function and mass was performed using Argus post-processing software (Siemens Healthineers). The systolic circumferential strain and diastolic strain rate parameters were calculated using feature tracking software Circle, Cardiovascular (cvi42®, Circle Cardiovascular Imaging Calgary, Canada) from the short-axis bSSFP cine images. The epicardial and endocardial borders were traced manually at diastole and the software then tracked the deformation throughout the cardiac cycle generating values for global circumferential strain and circumferential diastolic strain rate. In case of insufficient tracking of the endocardial and epicardial borders, contours were manually corrected and the tracking repeated.

### Transthoracic echocardiography

Transthoracic echocardiography (echo) was performed using a commercial echocardiographic system (iE33, Philips Healthcare, Best, The Netherlands). Measurements of LV diastolic function were performed according to the guidelines [24]. The following diastolic indices were obtained; transmitral early (E) and late (A) diastolic velocities, mitral annular early (e’) diastolic velocity, with calculation of E/A and E/e’ ratios.

### Cardiopulmonary exercise test (CPET)

Cardiopulmonary exercise testing was undertaken using an upright treadmill or bicycle ergometer protocol with simultaneous respiratory gas analysis after performing a spirometry, as described [16]. An incremental ramp protocol was utilised whereby speed and inclination (for treadmill) or resistance and speed (for bicycle exercise) were gradually increased with continual heart rate, blood pressure and ECG recording. All subjects were exercised to volitional fatigue, with a corresponding adequate respiratory exchange ratio (RER) achieved as a requirement for satisfactory effort defined as RER of > 1. Maximal oxygen consumption (VO_2_ max) was determined by averaging VO_2_ measures over 30 s of peak exercise.

### Statistical analysis

A priori sample size calculation was performed which was based on a change in PCr/ATP ratio. With a power of 80% and *p* < 0.05, a sample size of 18 would be required to identify a difference of 0.39 in PCr/ATP. As the true effect size for MTG was unknown in this population, a post-hoc analysis using MTG was performed which showed that 30 subjects (20 HFpEF and 10 controls) would have a power of 86%. Data are expressed as mean ± SD for description of study cohorts and mean ± SE for CMR and CMRS variables. Categorical data are presented as numbers and percentages. Comparisons between the two groups were performed by non-parametric method due to small sample size. The Chi-squared test or Fisher’s exact test were used to compare categorical data as appropriate. Bivariate correlations were performed in all subjects using Pearson’s or Spearman’s method as appropriate. Non-normally distributed data were log transformed to construct normal data. Variables with significant correlations with diastolic strain rate and VO_2_ max were entered into a stepwise multivariate model to determine predictors of diastolic strain rate and VO_2_ max. A *P*-value < 0.05 was considered significant. All statistical analyses were performed with SPSS (version 21, International Business Machines, Armonk, New York, USA).

## Results

### Description of patient cohort and baseline characteristics

Sixty-five patients with chronic breathlessness under follow up in the cardiology clinics were identified and screened in addition to 7 patients who were recruited by posters. Of these, 37 were eligible to enter the study, of which the first 31 consecutive patients took part in the study. Four patients were excluded due to incomplete study protocol. None of the patients had prior HF admissions, prior insults such as chemotherapy or radiation. None of the patients had been diagnosed or suspected to have amyloidosis, and CMR did not show findings suggestive of amyloidosis. Patients with history of angina, previous myocardial infarction or coronary revascularization were excluded. Although coronary angiography was not performed prior to entering the study, none of the patients had a history of angina, and they did not have angina or develop any ischaemic changes during CPET.

Table 1 shows demographic, clinical, echocardiographic, biochemical and VO_2_ max results for both HFpEF and healthy control groups. Both groups were matched with regards to age, gender, BMI and glucose and blood cholesterols. Frequency matching instead of individual (one-to-one) matching was performed which was based upon proportion of the age and gender for both cohorts. It was challenging to achieve equal number of controls to the HFpEF group, as a significant number of healthy elderly subjects had to be excluded due to concomitant medical problems. Although the age (72 vs 69) and gender (67 vs 57%) were not perfectly matched, the differences are numerically small (3 years for age and 10% for gender), and statistically they were not significant (*p* > 0.05). All HFpEF patients had (1) signs or symptoms of HF, (2) normal LVEF and LV cavity size, (3) evidence of mild diastolic dysfunction on Doppler echocardiography, and, additionally (4), objective evidence of cardiac cause of exercise limitation on CPET. We decided to use the latter as an additional inclusion criterion, as this provided evidence of a cardiac cause of exercise intolerance in our mild HFpEF population, rather than being attributed to physical deconditioning, commonly seen in healthy elderly [16, 25, 26], and the predicted values are entirely consistent with a meta-analysis of studies from which reference values were developed [27].

**Table 1:** Clinical, echocardiographic and biochemical characteristics

	HFpEF (*n* = 27)	Healthy Controls (*n* = 14)	*P* value
Age (years)	72 ± 7	69 ± 6	0.09
Female, *n* (%)	18 (67)	8 (57)	0.55
NYHA class, *n* (%)			
I	0	14 (100)	< 0.001
II	24 (89)	0	< 0.001
III	3 (11)	0	< 0.001
Body mass index (kg/m^2^)	29 ± 6	26 ± 5	0.10
Hypertension, *n* (%)	12 (44)	0	0.04
Atrial fibrillation, *n* (%)	0	0	–
Beta blockers, *n* (%)	3 (11)	0	0.53
ARB/ACE inhibitor, *n* (%)	14 (52)	0	0.004
Diuretics, *n* (%)	12 (44)	0	0.005
Statins, *n* (%)	11 (41)	0	0.02
Systolic BP (mmHg)	144 ± 26	132 ± 4	0.15
Diastolic BP (mmHg)	81 ± 12	80 ± 13	0.86
Heart rate (bpm)	68 ± 12	60 ± 12	0.10
E/A ratio	0.69 ± 0.23	0.86 ± 0.24	0.042
E/e’ ratio	10.87 ± 2.61	7.47 ± 2.45	< 0.001
LA size (ml/m^2^)	31.1 ± 15.4	16.9 ± 6.4	0.003
VO_2_ max (ml/min/kg)	17.7 ± 3.3	27.8 ± 7.7	0.004
Blood glucose (mmol/L)	5.4 ± 0.9	4.8 ± 0.4	0.07
Free fatty acids (mmol/L)	0.49 ± 0.28	0.54 ± 0.19	0.10
Triglycerides (mmol/L)	1.26 ± 0.37	1.13 ± 0.45	0.44
LDL (mmol/L)	2.45 ± 0.71	3.17 ± 1.05	0.03
HDL (mmol/L)	1.52 ± 0.41	1.60 ± 0.41	0.59
BNP (pmol/L)	15.6 ± 9.4	6.8 ± 3.8	0.01

Values are mean ± SD or percentages

*ACE* Angiotensin-converting enzyme-inhibitors, *ARB* Angiotensin-receptor antagonist-II, *BNP* Brain natriuretic peptide, *HDL* High-density lipoprotein, *HFpEF* Heart failure with preserved ejection fraction, *LDL* Low-density lipoprotein

### Assessment of left ventricular function and geometry

Table 2 summarises CMR results for both groups. When compared to controls, HFpEF showed concentric remodeling as indicated by increased LV mass to LV end diastolic volume (EDV) ratio but normal LV mass index. As expected diastolic strain rate in HFpEF was significantly impaired. Despite normal LVEF, peak systolic circumferential strain was significantly impaired in HFpEF, indicating additional subtle contractile dysfunction. None of the HFpEF or controls had a late gadolinium enhancement (LGE) pattern indicating previous myocardial infarction. To assess gender difference in LV functional parameters, analyses were performed comparing LVEDV, LV end systolic volume (ESV) and LVEF between women and men separately in both groups. There were no significant gender differences in LV volumes and function in both groups (results not shown).

**Table 2:** CMR and CMRS results

	HFpEF (*n* = 27)	Healthy Controls (*n* = 14)	*P* value
PCr/ATP ratio	1.60 ± 0.09	2.00 ± 0.10	0.005
Cardiac lipid/water (%)	1.45 ± 0.25	0.64 ± 0.16	0.009
Diastolic strain rate (%/s)	84.6 ± 5.3	110.4 ± 5.5	0.002
Systolic circumferential strain (%)	−19.5 ± 0.4	−21.8 ± 0.5	0.002
LV end-diastolic volume (ml/m^2^)	62 ± 2	66 ± 4	0.20
LV end-systolic volume (ml/m^2^)	18 ± 2	20 ± 2	0.40
LV stroke volume (ml)	87 ± 3	92 ± 6	0.38
LV ejection fraction (%)	72 ± 1	70 ± 2	0.48
LV wall thickness (mm)	13 ± 1	9 ± 1	< 0.001
LV mass (g)	101 ± 5	90 ± 6	0.22
LV mass index (g/m^2^)	54 ± 3	50 ± 3	0.39
LV mass/EDV (g/mL)	0.86 ± 0.05	0.68 ± 0.05	0.03

Values are mean ± SE

*EDV* End-diastolic volume, *LV* Left ventricular, *PCr* Phosphocreatine, *ATP* Adenosine triphosphate

### Assessment of cardiac metabolism with ^1^H- and ^31^P cardiovascular magnetic resonance spectroscopy

**Fig. 1:**
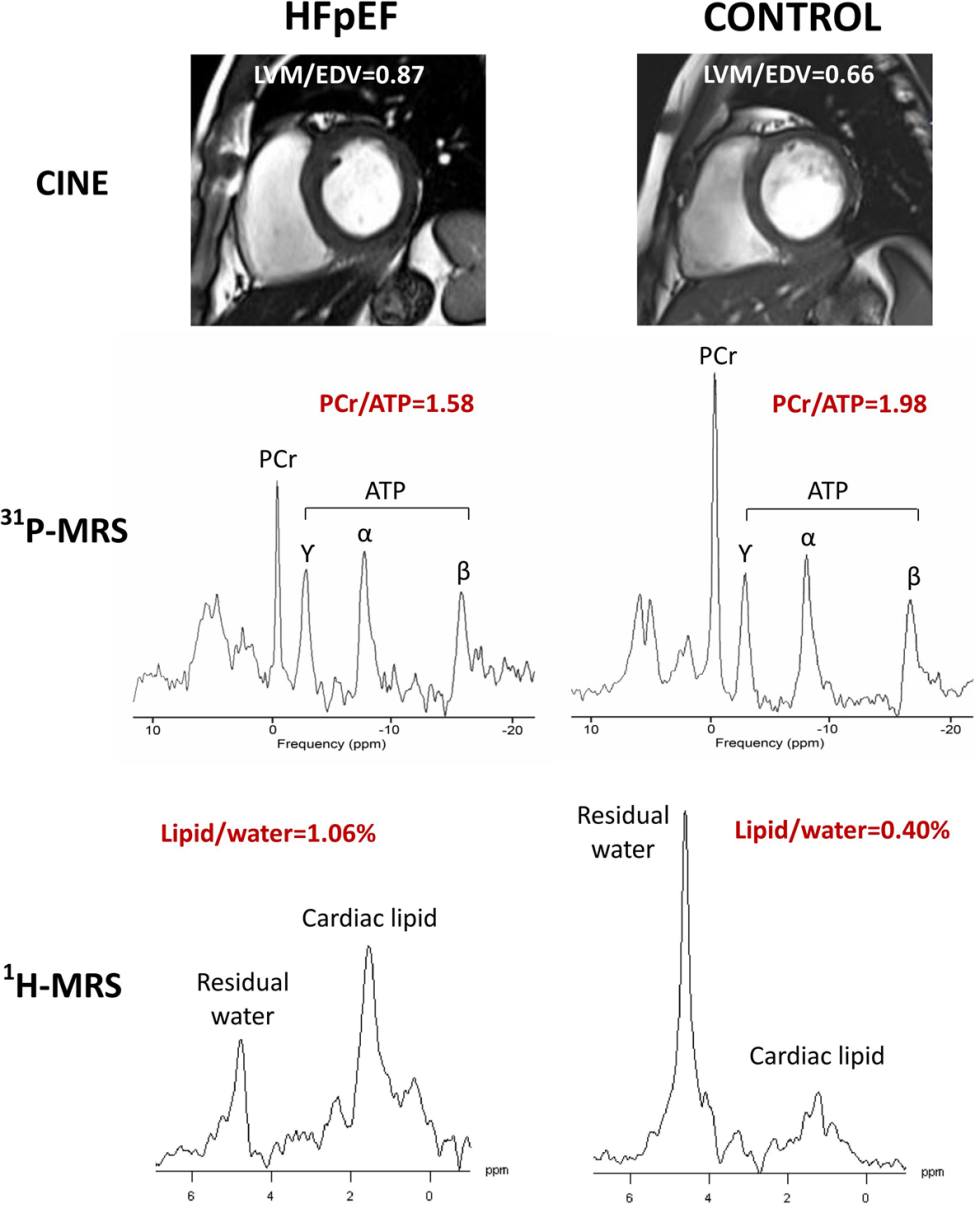
Cine imaging (top panel), ^31^P-CMRS (middle panel) and ^1^H-CMRS (bottom panel) showing representative results of LVM/EDV, PCr/ATP and Lipid/water for heart failure with preserved ejection fraction (HFpEF) (left) and control (right). ^1^H-CMRS spectra are scaled based on unsuppressed water (not shown) and noise level. LVM = left ventricular mass; EDV = end-diastolic volume; CMRS = cardiovascular magnetic resonance spectroscopy

Table 2 also shows the comparison of ^1^H and ^31^P CMRS results for all study groups. Compared to controls, HFpEF patients had pronounced steatosis (a 2.3-fold increase) and impaired energetics despite similar age, gender and BMI (Fig. 1).

### Myocardial triglyceride, energetics, diastolic strain rate and VO_2_ max

**Fig. 2:**
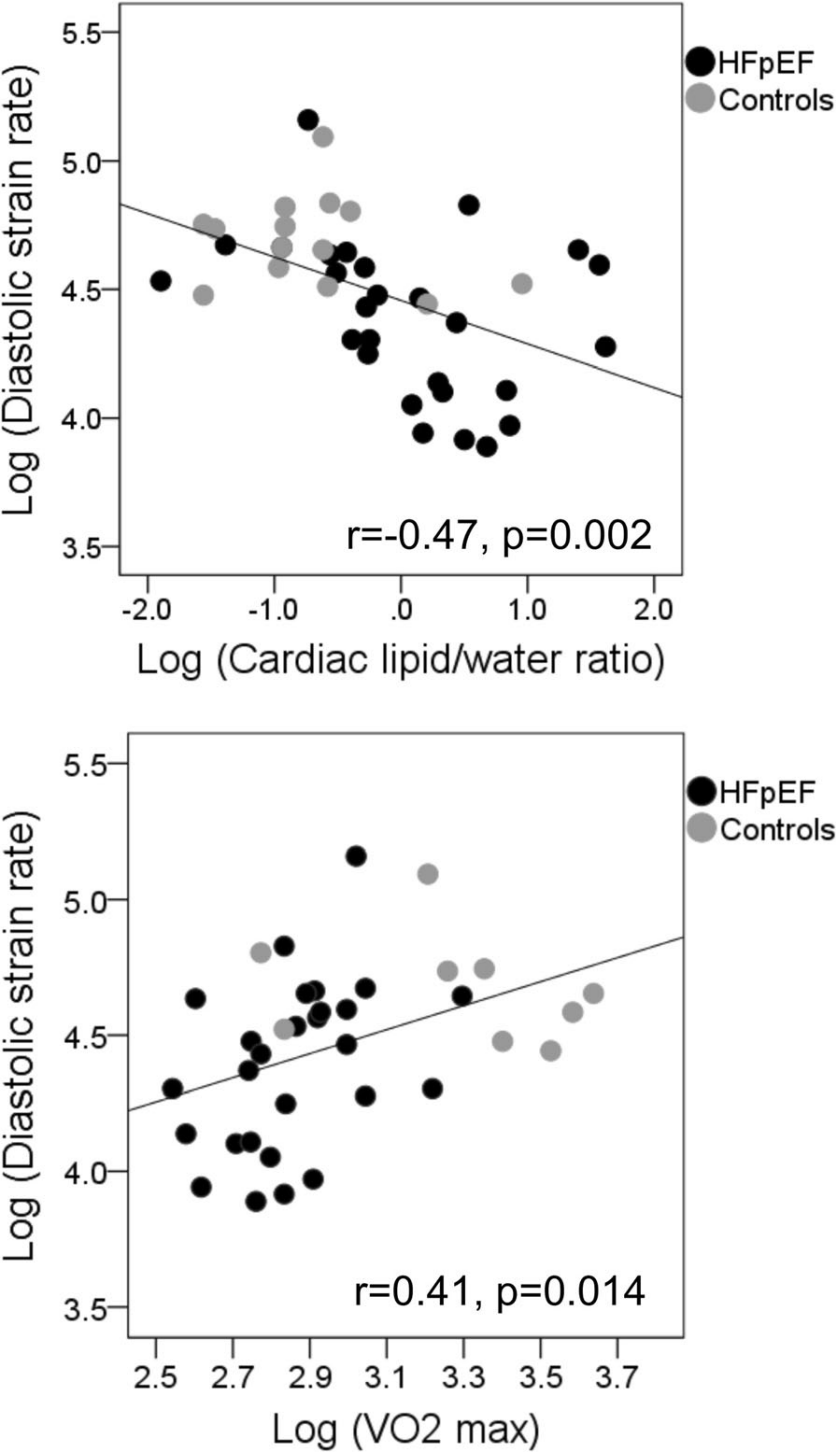
Scatter plot diagrams showing significant correlations between diastolic strain rate and cardiac lipid/ratio, and with maximal oxygen consumption (VO2 max)

Elevated MTG (Fig. 2) and reduced PCr/ATP ratio correlated significantly with impaired diastolic strain rate but no significant correlations with other diastolic indices such as transmitral E/A or E/e’. There were no significant correlations observed between MTG and PCr/ATP with echo diastolic indices, as the echo variables were not powered to detect these differences. On stepwise multivariate regression analysis, MTG but not PCr/ATP independently correlated with diastolic strain rate (adjusted R^2^ = 0.48) (Table 3). Increased MTG, reduced PCr/ATP, and impaired diastolic strain rate (Fig. 2) correlated significantly with reduced VO_2_ max but on stepwise multivariate analysis only diastolic strain rate was an independent correlate of VO_2_ max (adjusted R^2^ = 0.30). As expected both MTG (*r* = 0.35, *p* = 0.027) and diastolic strain rate (*r* = − 0.48, *p* = 0.001) correlated significantly with New York Heart Association (NYHA) class suggesting there may be a relationship between cardiac lipid and exercise tolerance.

**Table 3:** Bivariate correlations and multivariate correlations for diastolic strain rate and VO_2_ max

	Bivariate		Multivariate	
	R	*P*-Value	β	*P*-Value
Diastolic strain rate				
Myocardial triglyceride	−0.47	0.002	−0.58	0.001
PCr/ATP ratio	0.41	0.026	0.11	0.45
Age	−0.18	0.26	–	–
LVM/EDV	−0.20	0.30	–	–
SBP	−0.16	0.92	–	–
BNP	− 0.19	0.29	–	–
LA size	−0.10	0.59	–	–
VO_2_ max				
Myocardial triglyceride	−0.34	0.04	−0.35	0.23
Diastolic strain rate	0.41	0.014	0.57	0.007
PCr/ATP ratio	0.49	0.014	0.35	0.13
Age	−0.29	0.08	–	–
LVM/EDV	−0.33	0.11	–	–
SBP	−0.20	0.28	–	–
BNP	0.21	0.25	–	–
LA size	−0.21	0.28	–	–

*PCr* Phosphocreatine, *ATP* Adenosine triphosphate, *LVM* Left ventricular mass, *EDV* End-diastolic volume, *SBP* Systolic blood pressure, *BNP* Brain natriuretic peptide, *LA* Left atrial

By using the cut off values of abnormal lipid/water > 0.64 and abnormal PCr/ATP of < 2.0 from the current study, of the 27 HFpEF patients, 21/27 (78%) had abnormal lipid/water and 17/20 (85%) had abnormal PCr/ATP. Of the 14 controls, 3/14 (21%) had abnormal lipid and 4/10 (40%) had abnormal PCr/ATP and the rates of these abnormalities between HFpEF and controls were statistically significant (*p* < 0.05). Furthermore, the patients with abnormal lipid/water and PCr/ATP ratio had significantly lower LV diastolic strain rate and VO_2_ max than those with normal lipid/water and PCr/ATP ratio (results not shown). By using the cut-off values of abnormal lipid/water > 0.77 and abnormal PCr/ATP < 1.6, of the 27 HFpEF patients, 19/27 (70%) had abnormal lipid/water and 15/20 (75%) had abnormal PCr/ATP.

In line with our previous work [13], we found that MTG had a significant correlation with peak systolic circumferential strain (*r* = 0.61, *p* < 0.001). In addition we found that reduced PCr/ATP correlated with impaired peak systolic circumferential strain (*r* = − 0.55, *p* = 0.002) but no significant correlations with age, BMI, systolic blood pressure (SBP) or BNP.

## Discussion

The present study has three major findings. First, there is pronounced myocardial steatosis in patients with mild HFpEF, with a 2.3-fold increase in MTG compared to age, gender- and BMI-matched healthy controls. Second, steatosis (but not energetics) is independently associated with impaired diastolic strain rate. Third, reduced VO_2_ max is related to elevated MTG, and this relationship may be mediated through impaired diastolic strain rate.

While steatosis is known to be associated with diastolic dysfunction in diabetes [28], data on steatosis in HFpEF and its relationship with cardiac function and exercise capacity are lacking. There is only one study recently showing steatosis in a small number of young women (*n* = 5) with microvascular dysfunction and subclinical HFpEF [15]. Here, we demonstrate for the first time MTG in a larger number (*n* = 27) of typically elderly patients with HFpEF, who have limiting exertional dyspnoea, abnormal diastolic function demonstrated by Doppler echo and CMR FT, and objective evidence of exercise-limitation by CPET. We categorized our HFpEF cohort as mild as although they fulfilled the criteria based on the guidelines [18, 19] their diastolic abnormalities were mildly impaired which were likely due to the effect of taking diuretics.

Importantly, we show that elevated MTG independently correlated with impaired diastolic strain rate. The mechanisms leading to such pronounced steatosis remain to be completely understood, but it is well established that cardiac hypertrophy is associated with altered myocardial substrate metabolism with a shift towards glucose and away from FA oxidation, leading to increased myocardial lipid accumulation, non-oxidative metabolism and reduced cardiac function [8, 29, 30]. While our data show that MTG and diastolic strain rate have significant correlations with exercise capacity, only diastolic strain rate independently correlated with VO_2_ max. This is not surprising given the fact the abnormal resting diastolic function has been shown to be associated with worsening of exercise capacity [31]. Furthermore the underlying mechanism of MTG directly affecting VO_2_ max is unclear and could potentially be mediated through reduced diastolic strain rate. While our data do not prove a direct causal link, they may suggest a pathophysiological role of steatosis in the development of diastolic dysfunction and reduced functional capacity in mild HFpEF. It would have been interesting to demonstrate more profound impairment in diastolic function along with changes in metabolic substrate metabolism during exercise. In fact, Phan et al has previously shown more profound diastolic abnormalities during stress radionuclide ventriculography in HFpEF, which were not seen at rest [16]. In future, taking blood samples for plasma substrate and metabolomics during exercise to assess changes in substrate metabolism would support the findings in the present study.

In the present study, our HFpEF patients had increased LV wall thickness and significant concentric remodeling, despite normal LV mass. Given our cross-sectional study design, we cannot determine if cardiac steatosis causes LV remodeling or vice versa. However, several studies using animal models of pathological hypertrophy have demonstrated a causal link between steatosis and development of cardiac remodeling [29, 30]. Cardiac steatosis can lead to production of harmful intermediates and apoptosis. These can stimulate hypertrophic signalling leading to concentric LV hypertrophy followed by eventually a dilated phenotype [32, 33]. The question remains, what is the potential driving factor for metabolic alteration and steatosis in HFpEF? Kato et al recently showed that HFpEF patients have reduced coronary reserve due to microvascular dysfunction [34], and this has been proposed as a possible trigger of the metabolic switch and steatosis prior to the development of cardiac hypertrophy [15].

Impaired myocardial energetics has been demonstrated in asymptomatic diastolic dysfunction due to obesity [35] and also in HFpEF [16]. Here we extend the findings by showing significant correlations between energetics, diastolic strain rate and exercise capacity in mild HFpEF. Metabolic alterations in hypertrophied hearts have been shown to include impaired myocardial energetics [30], and detrimental effects of ceramides on mitochondria can lead to reduced intracellular ATP production, apoptosis and reduced cardiac function [36]. This may be a mechanism behind the observed relationship between myocardial energetics and function in the current study. Although both myocardial energetics status and triglyceride content correlated with diastolic strain rate, only steatosis independently correlated with diastolic strain rate. These are in keeping with a previous study in patients with type 2 diabetes mellitus and preserved LVEF, showing an independent association of steatosis with diastolic dysfunction [28]. This is an important finding and suggests that the pathophysiological cascade leading to diastolic dysfunction in HFpEF may involve steatosis at an earlier stage than energetic derangement – an observation with potential consequences in our search for therapeutic targets in HFpEF.

The current study provides novel insight into the pathophysiological role of steatosis in mild HFpEF. While inhibition of FA oxidation and stimulation of glucose oxidation may be beneficial in HF due to ischaemic insults [37, 38], cardiac lipid modulation by augmenting FA oxidation might be an alternative therapeutic strategy in non-ischaemic HF [8, 30]. Since myocardial steatosis is modifiable, novel metabolic therapies aimed at improving/preserving cardiac function and exercise capacity, thus delaying the progression to the more severe form of HFpEF by reducing MTG should be tested. Potential therapeutic agents are glucagon-like peptide-1 receptor (GLP-1) agonists, mineralocorticoid receptor blockers such as eplerenone, and fenofibrate, which have been shown to reduce myocardial steatosis in Type 2 Diabetes [33, 39, 40].

### Study limitations

As this study is limited by a small sample size, further corroboration of these findings in larger-scale multi-centre studies is required. Although the current study is powered to detect a difference, it may be underpowered to confirm the null hypothesis. Therefore, the non-statistical age difference between control and HFpEF cohorts would imply that age could still be a confounding variable for the difference in MTG observed. However, it would not be physiologically possible for a small age difference to result in > two-fold increase in MTG. In the present study, we excluded patients with a history of coronary artery disease and there was no evidence of myocardial infarction on CMR LGE imaging. Myocardial stress perfusion to assess microvascular dysfunction was not performed, as we did not want to burden these frail patients with a longer scan protocol. Thus, reduced coronary microvascular reserve during stress cannot be excluded as a potential mechanism contributing to the metabolic alteration in HFpEF. Due to the relatively long scan protocol, it was not feasible to perform reproducibility tests in the current study, thus reproducibility of lipid/water and PCr/ATP techniques in HFpEF is unknown. However, the reproducibility of these techniques have been shown to be excellent in healthy subjects [11, 20]. The current study is not representative of the HFpEF population as patients with diabetes and uncontrolled hypertension were excluded. In the future, the analysis should be repeated in a larger sample size including patients with these comorbidities. As there is lack of accepted standard MTG normal threshold, the interpretation of the results may be limited as the findings are based on the normal MTG value at our institution. The relatively low MTG (0.64) in controls could reflect the healthiness of the cohort control that is not representative of normal aging. Future studies will be needed to investigate the true MTG in old sedentary individuals. Similarly, the participation of very healthy subjects cannot be excluded as a potential factor contributing to the supra normal PCr/ATP. Although results vary slightly between centres, the mean PCr/AT*P* value for healthy controls in the current study is consistent with the values reported previously in this age group [16, 41]. Finally, the observational nature of our study precludes inferences of attribution. Further interventional research is needed to examine the change in MTG or PCr/ATP to confirm causal relationship between diastolic dysfunction/VO_2_ max and metabolic abnormalities.

## Conclusions

Mild HFpEF is characterized by pronounced myocardial steatosis, impaired myocardial energetics, impaired diastolic strain rate and reduced VO_2_ max. Reduced VO_2_ max may be attributable to elevated MTG via reducing diastolic function. MTG is a promising therapeutic target in HFpEF, thereby potentially improving exercise capacity, and, ultimately, outcomes in this difficult-to-treat patient population.

## Abbreviations

ATP: Adenosine triphosphate
BMI: Body mass index
BNP: Brain natriuretic peptide
bSSFP: Balanced steady state free precession
CMR: Cardiovascular magnetic resonance
CMRS: Cardiovascular magnetic resonance spectroscopy
CPET: Cardiopulmonary exercise test
CSI: Chemical shift imaging
ECG: Electrocardiogram
Echo: Echocardiogram
EDV: End diastolic volume
EF: Ejection fraction
ESV: End systolic volume
FA: Fatty acid
FT: Feature tracking
HF: Heart failure
HFpEF: Heart failure with preserved ejection fraction
LA: Left atrium/left atrial
LGE: Late gadolinium enhancement
LV: Left ventricle/left ventricular
MTG: Myocardial triglyceride
PCr/ATP: Phosphocreatine-to-adenosine triphosphate
RER: Respiratory Exchange ratio
SBP: Systolic blood pressure
STEAM: Stimulated echo
TE: Echo time
TR: Repetition time
VO_2_ max: Maximal oxygen consumption
